# Functional characterization of *CNOT3* variants identified in familial adenomatous polyposis adenomas

**DOI:** 10.18632/oncotarget.27003

**Published:** 2019-06-11

**Authors:** Richard Glenn C. Delacruz, Imelda T. Sandoval, Kyle Chang, Braden N. Miller, Laura Reyes-Uribe, Ester Borras, Patrick M. Lynch, Melissa W. Taggart, Ernest T. Hawk, Eduardo Vilar, David A. Jones

**Affiliations:** ^1^ Functional and Chemical Genomics, Oklahoma Medical Research Foundation, Oklahoma City, OK, USA; ^2^ Department of Clinical Cancer Prevention, The University of Texas UTHealth MD Anderson Cancer Center, Houston, TX, USA; ^3^ Department of Gastroenterology, Hepatology and Nutrition, The University of Texas UTHealth MD Anderson Cancer Center, Houston, TX, USA; ^4^ Department of Pathology, The University of Texas UTHealth MD Anderson Cancer Center, Houston, TX, USA; ^5^ Department of GI Medical Oncology, The University of Texas UTHealth MD Anderson Cancer Center, Houston, TX, USA; ^6^ Clinical Genetics Program, The University of Texas UTHealth MD Anderson Cancer Center, Houston, TX, USA; ^7^ Graduate School of Biomedical Sciences, The University of Texas UTHealth MD Anderson Cancer Center, Houston, TX, USA; ^8^ College of Medicine, University of Oklahoma Health Sciences Center, Oklahoma City, OK, USA

**Keywords:** familial adenomatous polyposis, colon cancer, APC, CNOT3, CtBP1

## Abstract

Germline mutations in the tumor suppressor *Adenomatous Polyposis Coli* (*APC*) define Familial Adenomatous Polyposis (FAP), the genetic predisposition to developing adenomatous polyps. Recent sequencing of FAP adenomas have challenged established dogma that *APC* mutations alone represent the adenoma mutational landscape because recurrent somatic mutations in non-WNT pathway genes were also discovered. In particular, one of these novel genes, *CNOT3*, presented E20K and E70K mutations that are predicted to be deleterious *in silico*. We utilized zebrafish embryos to determine if these mutations affect *CNOT3* function and perform novel biology in an APC-dependent pathway *in vivo*. Human *CNOT3* (*hCNOT3*) and *E20K* mRNA injection rescued zebrafish *cnot3a* knockdown lordosis phenotype while *E70K* did not. In the FAP *apc^mcr^* zebrafish model, we show that *ctbp1*, but not retinoic acid, regulates *cnot3a* expression. Injection of *hCNOT3* and *E20K*, but not *E70K*, to homozygous *apc^mcr^* zebrafish initiated gut differentiation while *cnot3a* knockdown in wildtype embryos led to decreased intestinal development and differentiation. Finally, targeted sequencing of 37 additional FAP adenomas revealed *CNOT3* mutations in 20% of these samples. Overall, our work supports a mechanism where *CTBP1* regulates *CNOT3* and that overall *CNOT3* perturbation could work in concert with germline *APC* mutations in advancing adenomas to a more transformed state prior to progression to adenocarcinoma.

## INTRODUCTION

Adenomatous Polyposis Coli (APC) is a 312 kDa protein that functions as a tumor suppressor by acting as a requisite scaffolding protein that stabilizes the β-catenin destruction complex. Somatic mutations in the *APC* gene are the most prevalent initiating event in colorectal carcinogenesis [1, 2]. These deleterious *APC* mutations mostly occur in a specific region, the *APC* mutation cluster region (MCR), and singularly define Familial Adenomatous Polyposis (FAP), a genetic condition predisposing to the development of colorectal adenomatous polyps and early onset colorectal adenocarcinoma [3, 4]. Mechanistically, these *APC* mutations are thought to disrupt the role of *APC* in promoting β-catenin degradation to temper proliferative canonical Wnt signaling [1, 5], and in modulating retinoic acid-dependent intestinal development [6–9]. Over time, additional genetic insults to cancer driver genes *KRAS* and *TP53* together with the underlying APC defect eventually progress FAP adenomas to colorectal cancer [10, 11].

*APC* is widely assumed as the only relevant gene mutated in early adenomas, but it is only recently that next-generation sequencing of colon adenomas have resulted in data to confirm this long-standing hypothesis [12, 13]. In our recent work to define a comprehensive genomic landscape of adenomas and at-risk mucosa, we found that patient-derived FAP adenomas do not just have the expected somatic *APC* mutations, but also recurrent mutations in *Wnt* pathway genes and in novel genes previously not linked to progressing colon adenomas to adenocarcinomas. Surprisingly, when the *APC* gene is excluded, one gene with frequent accumulating genomic alterations *CCR4-NOT Transcription Complex Subunit 3* (*CNOT3*), which was observed in 5 out of 25 sequenced FAP adenomas [13].

Transcription complexes are comprised of multiple proteins that perform combinatorial regulatory functions critical to proper transcription. The CCR4-NOT complex is a highly conserved, mRNA transcription regulator [14, 15] that functions mainly through the deadenylation and ubiquitination activity of its CCR4 (CNOT6) and NOT4 (CNOT4) subunits, respectively [16, 17]. CNOT3 is thought to primarily serve as a scaffolding protein in CCR4-NOT but there is growing evidence linking *CNOT3* mutations to disease [18, 19]. In the COSMIC database, the two most common *CNOT3* mutations in cancers are E → K mutations at amino acid positions 20 and 70, which are the *CNOT3* mutations we found in our adenoma samples [13, 20]. Unfortunately, there are no published work that details a unique mechanistic role for the NOT3 domain where the two E → K mutations are located to help guide us to the possible effects of these two mutations. A partial mechanistic role was reported by Suzuki et al that described a cooperative function for the N-terminal and C-terminal ends of CNOT3 in mRNA decay [21]. Overall, more work needs to be done to fully understand the mechanistic role of the NOT3 domain. Additionally, because the CCR4-NOT complex has numerous biological activities, the functional consequences of the CNOT3 E20K and E70K mutations will be very difficult to ascertain using traditional *in vitro* methods. To circumvent this, we turn to zebrafish and employ *in vivo* functional genomics analyses.

From our experiments, *cnot3* knockdown and *apc* deficiency are rescued by human CNOT3 and the E20K variant but not by E70K, suggesting that E70K is an inactivating mutation. We also provide mechanistic evidence that *CNOT3* is genetically linked to *APC* through *CtBP1*. Taken together with our findings that *CNOT3* mutations are present in approximately 20% of FAP adenomas, we conclude that proper *CNOT3* function is important for intestinal development and that *CNOT3* inactivation might work in concert with *APC* deficiency to prevent intestinal differentiation and potentially advance colon adenomas to a more transformed state.

## RESULTS

### CNOT3 E20K and E70K are common mutations in cancer tissues

**Figure 1:**
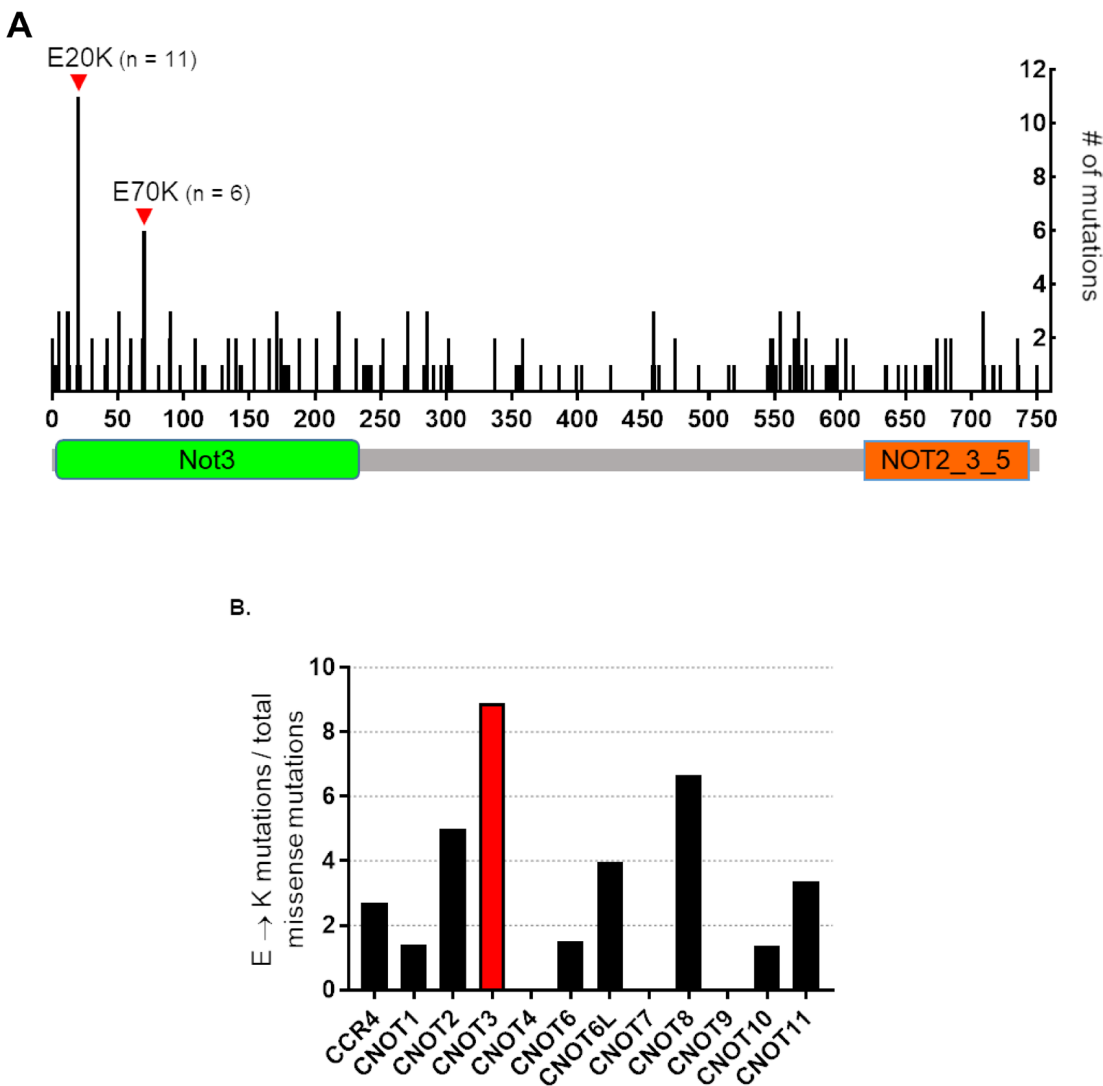
Human CNOT3 E to K mutations in cancer. (**A**) Schematic representation of CNOT3 protein showing the numerous mutations throughout the CNOT3 amino acid sequence in cancers. The two most common missense mutations are E20K and E70K, which occur in the Not3 domain (green box) and are the two mutations that we discovered during FAP adenoma sequencing experiments [13]. (**B**) Percentage of E to K mutations compared to total missense mutations in the twelve CCR4-NOT supercomplex subunits over all cancer samples in the cBIOPORTAL database. CNOT3 is presented in red.

We previously reported that *CNOT3* mutations occur in FAP adenoma [19]. To determine how prevalent these mutations are in cancer, we mined the publicly available cBIOPORTAL for Cancer Genomics database [19]. From our analyses, *CNOT3* alterations (mutations, deletions, and amplifications) are common across multiple types of cancers in sequenced patient and cell line tumor samples. Remarkably, the *CNOT3* E20K and E70K mutations we found in adenoma tissues are the two most common mutations found in numerous cancer types (Figure 1A). Additionally, of all the CCR4-NOT supercomplex subunits, the scaffold protein CNOT3 has the most number of E → K mutations (Figure 1B). These results suggest that the CNOT3 E → K variants identified in FAP adenomas are clinically relevant, thus justifying the need for their functional characterization.

### CNOT3 is required for intestinal differentiation

**Figure 2:**
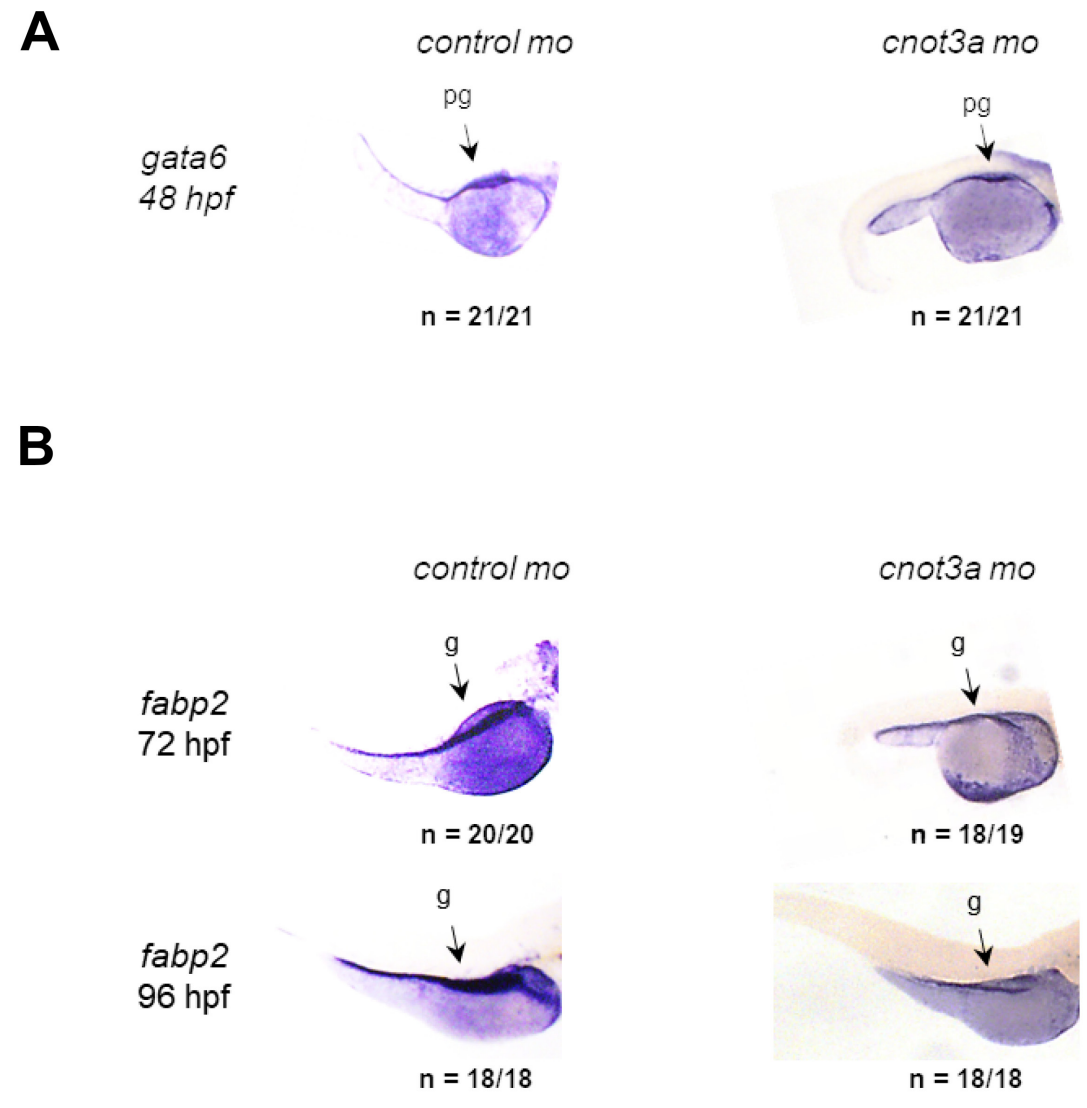
*CNOT3* is required for intestinal differentiation. WISH staining for (**A**) the gut precursor marker *gata6* (pg) at 48 hpf and (**B**) the differentiated gut-specific marker *fabp2* (g) in 72 and 96 hpf control and *cnot3a* morphants.

We used zebrafish embryonic development as an unbiased, whole organism readout for identifying the *in vivo* activity of CNOT3 and the functional consequences of the E20K and E70K mutations. We first characterized the relevant *CNOT3* orthologue in zebrafish to be *cnot3a* (See Supplementary Figures 1 and 2 and Supplementary Table 1). We then interrogated the involvement of *cnot3* in embryonic intestinal formation by staining *cnot3a* morphants for primordial intestine marker *gata6* and intestinal differentiation marker *fabp2*. At 48 hpf, when intestinal premordium (pg) is already present, *gata6* staining is comparable in *cnot3a* morphants and control group (Figure 2A). From 72 hpf to 96 hpf, *cnot3a* morphants exhibited minimal increase in *fabp2* (g) staining while control embryos have significantly increased staining from one timepoint to the next (Figure 2B). Our results show that *cnot3a* knockdown hinders intestinal differentiation and suggest that proper *cnot3a* expression plays a role in this important developmental process.

### CNOT3 and E20K rescue cnot3a depletion but E70K variant does not

**Figure 3:**
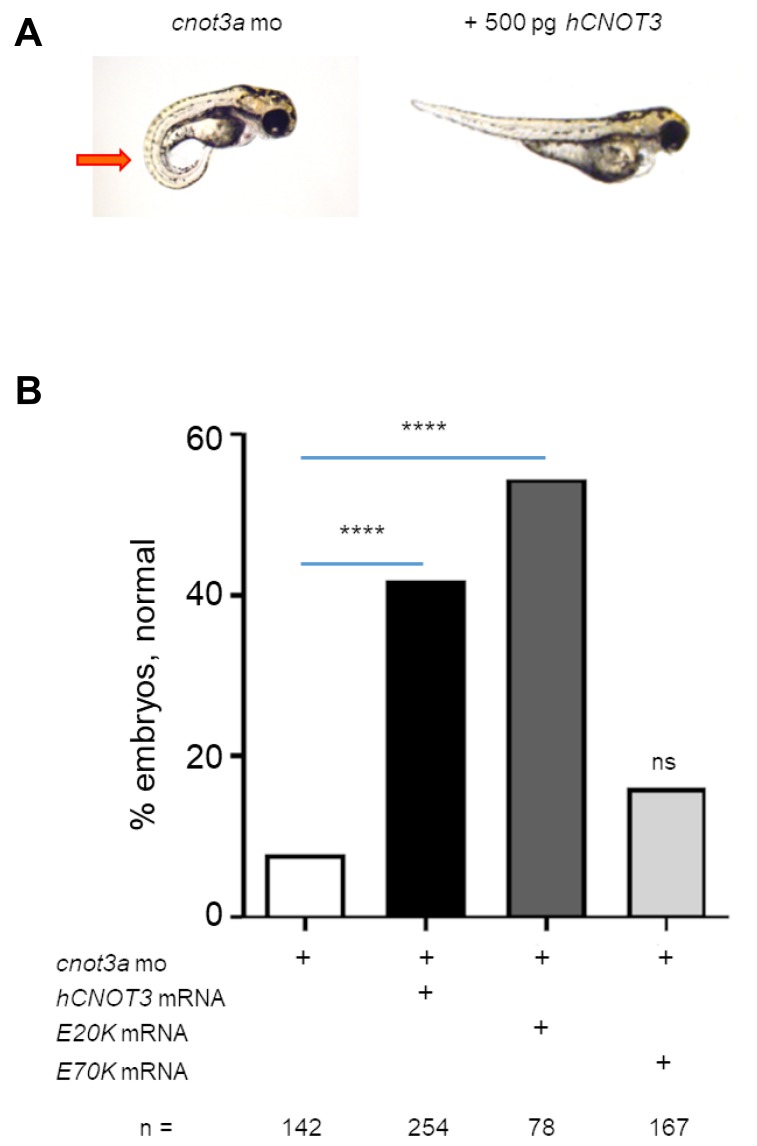
Functional characterization of CNOT3 variants. (**A**) The lordosis phenotype (red arrow) caused by *cnot3a* knockdown was rescued by co-injection of 250 pg wildtype human *CNOT3* (*hCNOT3*) with 4 ng *cnot3a* morpholino (mo). (**B**) Percentage of rescue after co-injection of *hCNOT3, E20K,* or *E70K* variants with *cnot3a* mo (*n* > 75 per group). Significance of co-injection rescue was determined using Fisher’s exact test compared to *cnot3a* mo injection alone. ns = not significant. ^****^= *p*-value < 0.0001.

To validate that human *CNOT3* function translates into the zebrafish, we co-injected *CNOT3* mRNA with *cnot3a* morpholino in 1-2 cell stage embryos and used the highly penetrant lordosis phenotype caused by *cnot3a* knockdown (Supplementary Figure 2) as a readout for complementation. Our results show that the co-injected group had significantly fewer embryos with the lordosis phenotype compared to the *cnot3a* morpholino-only group, thus confirming that wildtype *CNOT3* compensates for *cnot3* knockdown (Figure 3A and 3B).

Next, we determine whether the two *CNOT3* mutations identified in FAP adenomas [13] affect wildtype *CNOT3* function *in vivo* by co-injecting each one with *cnot3a* mo into 1-2 cell stage zebrafish embryos. Our results show that *CNOT3 E20K* variant rescues the observed *cnot3a* morphant lordosis phenotype significantly while *E70K* does not (Figure 3B). Our data suggests that *CNOT3 E70K* is an inactivating mutation.

### Human CNOT3 E70K variant cannot rescue intestinal differentiation in the apc-deficient zebrafish model (apc^mcr^)

**Figure 4:**
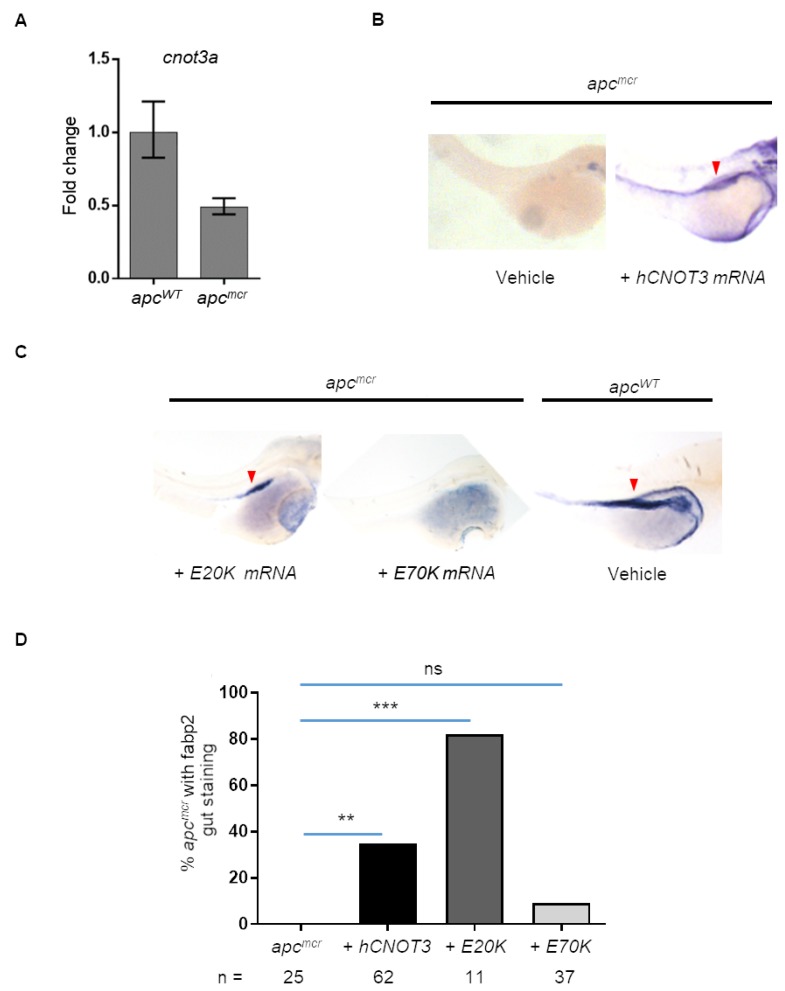
Human CNOT3 rescues intestinal defects in zebrafish apc^mcr^ embryos. (**A**) Graph shows *cnot3a* mRNA expression relative to *18s* in 72 hpf embryos. (**B**) *apc^mcr^* and wildtype embryos, WISH staining for gut marker *fabp2* for vehicle and *hCNOT3* injected *apc^mcr^* embryos. (**C**) WISH staining for *hCNOT3 E20K* and *E70K*-injected *apc^mcr^* embryos and vehicle-injected apc^WT^. Note that the red arrowhead = *fabp2* staining. (**D**) Percentage of *apc^mcr^* fish with gut marker staining from (A and B) at 72 hpf (*n* > 10 per group). Statistical significance of *CNOT3* mRNA injection rescue was determined using Fisher’s exact test compared to uninjected *apc^mcr^* embryos alone. ns = not significant; ^***^ = *p*-value = 0.0003; ^**^ = *p*-value = 0.0043.

To determine the genetic relationship of *apc* and *cnot3*a, we measure *cnot3a* expression by qRT-PCR in apc mutant (*apc^mcr^*) and wildtype sibling zebrafish embryos. We find that *apc^mcr^* fish have decreased *cnot3a* expression compared to control (Figure 4A) suggesting that *apc* regulates *cnot3a*.

Based on this result, we wondered whether the intestinal differentiation defect of apc^*mcr*^ zebrafish could be rescued by human CNOT3 mRNA injection. The *apc^mcr^* zebrafish is an established tool for understanding the genetics of colon cancer progression, and using the restoration of intestinal differentiation in homozygous *apc^mcr^* embryos as a readout has helped previously to uncover novel genes downstream of *APC* [6–8, 22–24]. Thus, to find out if *cnot3a* functions downstream of *apc*, we injected *CNOT3* mRNA to 1-2 cell *apc^mcr^* embryos and screened for intestinal differentiation at 72 hpf. Using the intestinal development marker *fabp2*, we observe that introduction of *CNOT3* mRNA to *apc^mcr^* embryos does initiate intestinal development (Figure 4B).

We exploit the finding that CNOT3 rescues *apc^mcr^* intestinal differentiation to determine the functional consequences of *CNOT3 E20K* and *E70K* mutations by injecting CNOT3 E20K and E70K mRNA into 1 - 2 cell stage *apc^mcr^* zebrafish embryos. Similar to *CNOT3*, the *E20K* variant rescues intestinal differentiation but *E70K* injection does not (Figure 4C–4D). The a*pc^mcr^* intestinal differentiation rescue assay results provide additional evidence that E70K is an inactivating mutation. *CNOT3* mRNA injections were confirmed by PCR (Supplementary Figure 3).

### CNOT3 is regulated by transcriptional repressor CTBP1

**Figure 5:**
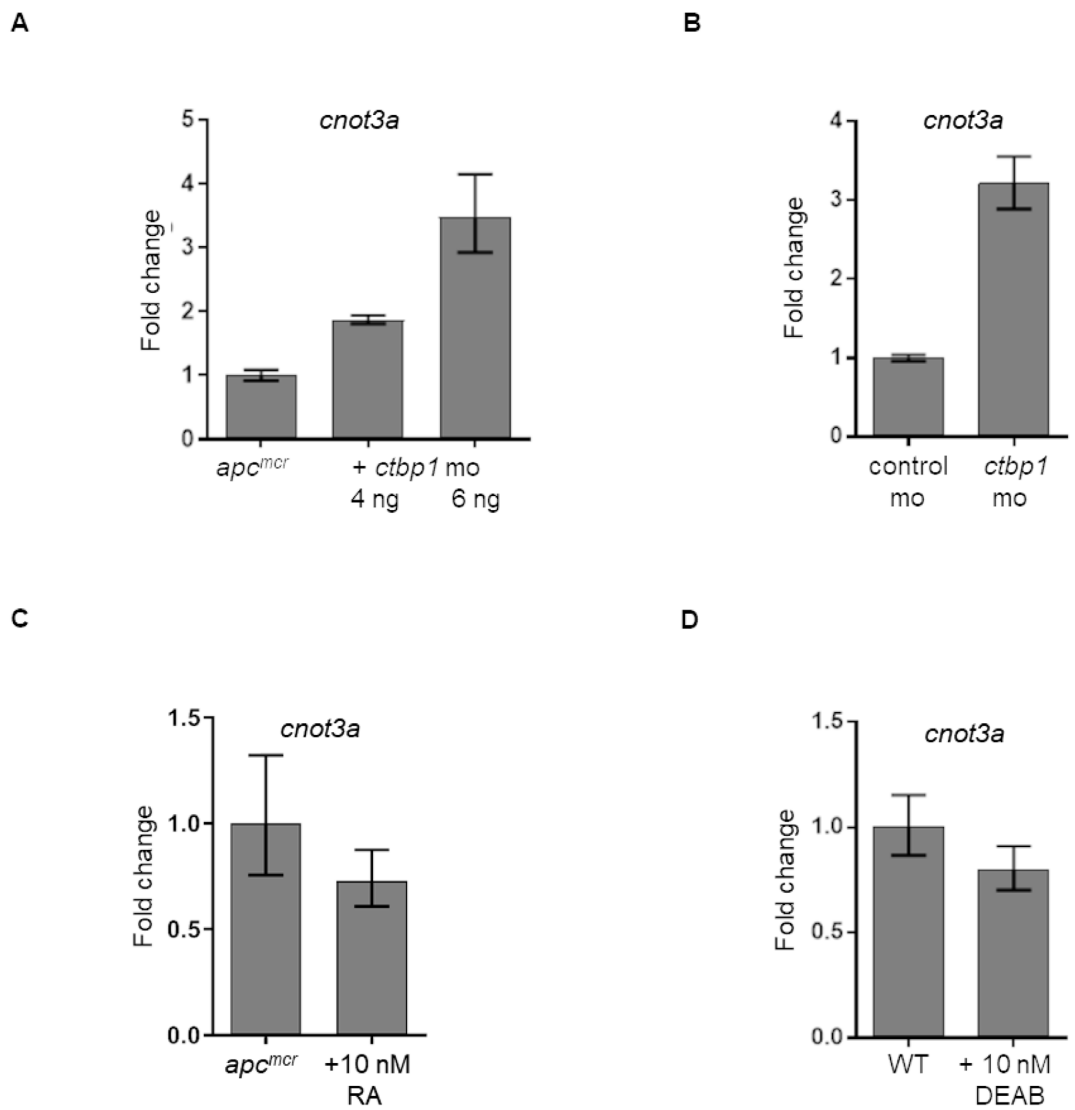
Regulation of cnot3a expression by ctbp1. (**A**–**D**) Graphs showing *cnot3a* mRNA expression relative to *18s* using qRT-PCR assay. mRNA expression of *cnot3a* in (A, C) *apc^mcr^* and (B, D) wildtype embryos after injection of *ctbp1* morpholino (mo) (A–B), and after exposure to (C) 10 nM retinoic acid (RA) or (D) RA inhibitor DEAB. Values represent mean ± SD. Graphs shown above is representative of 2 independent pooled embryo samples (3 technical replicates each).

Our data from the previous section suggests that *CNOT3* is downstream of *APC* during intestinal development (Figure 4A–4D). We have also shown elsewhere that *APC* regulates CtBP1 that in turn controls retinoic acid (RA) biosynthesis [6–8]. This led us to perform a series of qRT-PCR analyses to clarify how *CNOT3* fits in this model. We first explored how *ctbp1* knockdown, which we have previously shown to restore intestinal differentiation in *apc^mcr^* zebrafish [8], affects *cnot3a* levels. We observe that knockdown of the transcriptional corepressor *ctbp1* in both *apc*-deficient (Figure 5A) and *apc* wildtype (WT) (Figure 5B) embryos leads to a 2-3 fold increase in *cnot3a* expression suggesting that *cnot3a* is downstream of *ctbp1*. We have also previously reported that RA biosynthesis, which is regulated by *CTBP1,* partially rescues intestinal development [6–8]. To determine if *CNOT3* rescue of intestinal differentiation depends on RA biosynthesis, we exposed *apc* mutant fish to retinoic acid. Our qRT-PCR results show that *cnot3a* expression does not change with RA supplementation (Figure 5C). Similarly, exposure to the RA inhibitor, DEAB, also does not affect *cnot3a* expression (Figure 5D). These series of qRT-PCR experiments imply that CtBP1 regulates *CNOT3* independent of retinoic acid status.

**Figure 6:**
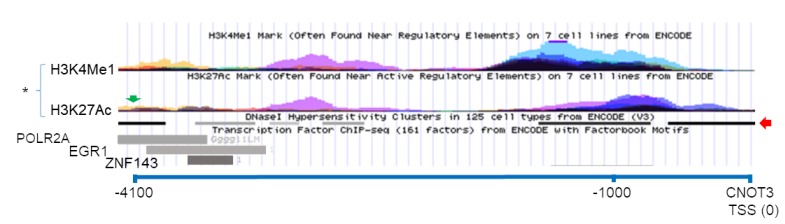
Possible *CTBP1* binding site near the *CNOT3* gene. USCS Genome Browser screen capture showing the CTBP1 binding site (green arrow) in chromosome 19 (-) 4100 bp of the CNOT3 Translational Start Site (TSS) from CTBP1-CHiP Seq dataset (33). (^*^) = H3K4Me1 and H3K27Ac signal from seven different high-throughput ENCODE cell line datasets. Red arrow show DNA regions with hypersensitivity to DNAse I treatment. Transcription Factor POLR2A, EGR1, and ZNF143 ChIP-Seq pulldown are represented as gray rectangles.

**Figure 7:**
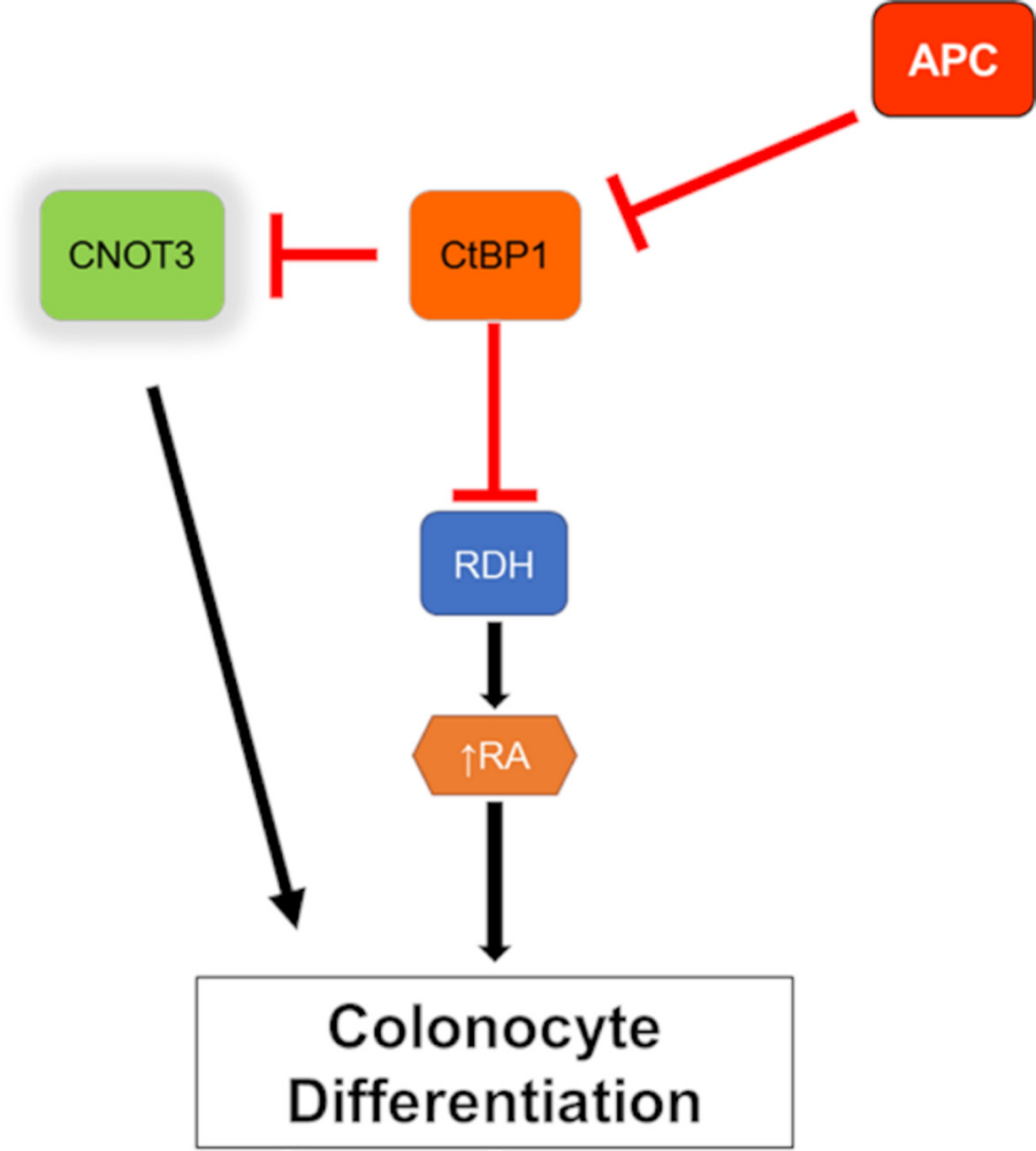
Model of *CNOT3* involvement in intestinal differentiation. Red line indicates inhibition and black arrow indicates activation.

A possible mechanism by which CTBP1 protein regulates *CNOT3* is by working as a transcriptional repressor of *CNOT3*. By mining a previously published CtBP1 CHiP-Seq dataset [25], we found evidence that CtBP1 binds approximately (-) 4,000 bp (chr19:54,637,229-54,637,258) of the *CNOT3* transcription start site (chr19:54,641,436) in chromosome 19 (Figure 6). Viewing ENCODE data using the UCSC Genome browser, this presumptive CtBP1 binding site possess characteristics of a transcription factor (TF) binding site including (i) DNase I hypersensitivity (31 / 125 cell types), (ii) RNA Polymerase II, EGR1, and ZNF143 CHiP-Seq pulldown, and (iii) H3K4Me1 and H3K27Ac signal [26–33]. Thus, we propose that CtBP1 binding to this site could help localize repression elements to suppress *CNOT3* expression. Taken together, our results suggest a novel mechanism wherein *APC* regulates *CNOT3* via *CTBP1* independent of retinoic acid (Figure 7).

### CNOT3 mutations are again present in an expanded cohort of fap adenoma samples

To confirm our previously published observation that five of twenty-five (20%) FAP adenoma samples carry *CNOT3* mutations [13], we further interrogated the mutational status of *CNOT3* in colorectal premalignancy by performing deep sequencing in all the exons using Ion Torrent (IT) in a new cohort of 37 adenomas of 14 FAP patients with paired germline samples (Supplementary Tables 2 and 3). The mean depth obtained for *CNOT3* from the IT was 5,047x. Seven adenomas (19%) harbored somatic *CNOT3* mutations, with the K286E mutation found in four of the samples (Table 1 and Supplementary Table 4). Comparing these *CNOT3* mutations against the sequenced tumor sample data in the COSMIC and cBIOPORTAL databases, we discovered that they are all novel mutations, thus expanding the spectrum of *CNOT3* alterations reported in tumor studies and confirming that approximately 20% of FAP adenomas have CNOT3 mutations.

**Table 1:** *CNOT3* mutations found in an extended cohort of FAP adenomas

Sample ID	Mutation
MDAC14_P05	E120A
MDAC34_P04	D285E
MDAC24_P02	K286E
MDAC29_P01	K286E
MDAC32_P01	K286E
MDAC34_P02	K286E
MDAC24_P01	Frameshift at G488

## DISCUSSION

The implementation of high throughput genomic sequencing has created a growing clinical need to streamline the evaluation of novel mutations in disease-causing genes for functional relevance and, therefore, their possible contribution to disease progress. Previous work on the *APC* tumor suppressor gene has shown it as being involved in numerous processes for normal development and homeostasis [34]. Central among these roles is *APC* control of intestinal differentiation and its ability to suppress turnover and proliferation of intestinal epithelia [35]. Although these roles are clearly the basis of *APC* function as the major driver of both sporadic and hereditary colon tumorigenesis [1–4], *APC* dysregulation alone though does not lead to colon cancer. Additional hits in other genes like *KRAS* and *TP53* are needed together with *APC* dysfunction to cause colon cancer [10, 11]. It is unclear, however, whether additional genes also contributed to tumorigenesis. Following up our previous work defining the genetic profile of adenomas and at-risk mucosa from FAP adenomas [13], here we use the zebrafish to rapidly functionally characterize two clinically-relevant *CNOT3* mutations and also offer evidence that *CNOT3* plays a critical role in intestinal differentiation downstream of *APC*.

Our work is the first to provide evidence that the *CNOT3 E70K* mutation, a common but previously uncharacterized mutation in cancers, is an inactivating mutation. Our work details two functional assays pointing to *CNOT3 E70K* being an inactivated variant (Figures 3 and 4). One plausible scenario based on the intestinal differentiation assay, is that the *CNOT3 E70K* inactivating mutation could be working with *APC* mutation to better prevent differentiation while waiting for other tumor-promoting mutations in *KRAS* and *TP53* to occur [10, 11]. Our data also hints of *CNOT3 E20K* being an activating mutation as it rescues both complementation and intestinal differentiation assays better than wildtype *CNOT3*. This was unexpected though not unprecedented as both gain-of-function and inactivating mutations in *TP53* have been reported to be tumor-promoting [36, 37]. Note that a limitation of our study is that we do not directly demonstrate that either *CNOT3 E20K* or *E70K* mutations are oncogenic. However, our study does create a rationale for studying *CNOT3* mutations in the context of adenoma progression to colon carcinoma.

We have previously shown that CtBP1 is downstream of APC and regulates RDHs during intestinal differentiation [6–8]. Based on our current findings that (i) *cnot3a* depletion leads to stalled intestinal differentiation (Figure 2), (ii) *apc* mutation in zebrafish results in decreased *cnot3a* expression (Figure 4A), (iii) *CNOT3* mRNA is able to restore differentiation of *apc^mcr^* intestine (Figure 4B), and (iv) *ctbp1* regulates *cnot3a* expression (Figure 5A, 5B) independent of RA biosynthesis (Figure 5C, 5D), we conclude that CNOT3 is connected to APC through CtBP1 and that CNOT3 works in parallel with retinoic acid to effect intestinal differentiation.

Finally, the prevalence of *CNOT3* mutations in the TCGA PanCancer dataset that contains 10,967 samples from 32 studies showed a Somatic Mutation Frequency (SMF) for *APC* of 7.3%, while *CNOT3* was 1.3% [19]. In this dataset, the Somatic Mutation Frequency (SMF) of *APC* is 7.3%, while *CNOT3* is 1.3%. In the colon cancer subset (594 samples), *APC* and *CNOT3* were altered in 66.67% and 1.18% of cancers, respectively. The percentage of *CNOT3* mutations (~20%) in FAP adenoma samples that we previously reported [13] and corroborated in this manuscript is higher compared to the TCGA data that contains only sporadic and inherited colon cancer samples. Since *CNOT3* mutations occur more favorably in FAP patients, our observations are very relevant in the context of personalized medicine and justifies the need to further characterize the novel “*CNOT3*-mutant FAP adenoma” subset in more detail.

## MATERIALS AND METHODS

### Zebrafish maintenance

Wild-type *TU* and *apc^WT/mcr^ Danio rerio* (zebrafish) were maintained as previously described [38]. Fertilized embryos were collected following natural spawnings in 1× E3 medium (286 mg/L NaCl, 13 mg/L KCl, 48 mg/L CaCl_2_·2H_2_O, 40 mg/L MgSO_4_, 0.01% methylene blue) and allowed to develop at 28.5° C.

### Morpholino and RNA microinjections

For RNA rescue experiments, full length human wildtype *CNOT3*, *E20K*, and *E70K* variant RNA transcripts were transcribed from linearized plasmid DNA using mMESSAGE mMACHINE transcription kit (ThermoFisher Scientific, Waltham, MA). For microinjections, 1-2 nl of RNA was injected with our without 4 ng *cnot3a* morpholino into embryos at the one-to-two cell stages. Overexpression of mRNA transcript was assessed by PCR. Statistical analyses were performed using Fisher’s exact test (GraphPad Prism v 7.02). Parental *CNOT3* plasmid was obtained from Origene (Rockville, MD).

A complete list of morpholinos and PCR primers used are provided in Supplementary Table 5.

*Zebrafish cnot3a and cnot3b in situ hybridization probes*. 400-bp *cnot3a* and *cnot3b* ORF gene fragments were chemically synthesized and attached to pUC57-Kan by Genewiz (South Plainfield, NJ). Sense and antisense probes were made using T3 and T7 DIG RNA Labeling kit (Roche, Basel, Switzerland), respectively, from linearized *cnot3a* and *cnot3b* pUC57 plasmids.

### *In situ* hybridization

Whole organism *in situ* hybridization (WISH) was performed as previously described using digoxigenin-labeled riboprobes for *gata6* and *fabp2 (fatty acid binding protein 2, intestinal)* [39]. Embryos were cleared in 2:1 benzyl benzoate / benzyl alcohol solution and documented using an Olympus SZX12/DP71 imaging system (Olympus Corporation, Shinjuku, Tokyo, Japan).

### Quantitative RT-PCR (zebrafish)

RNA from zebrafish embryo lysates was isolated using the RNeasy kit (Qiagen, Hilden, Germany). cDNA was synthesized from 1 μg of total RNA using iScript (Bio-Rad, Hercules, CA). Intron-spanning primers, when possible, were designed using the Universal ProbeLibrary Assay Design Center (Roche). A complete list of primer sets is provided in Supplementary Table 5.

PCR master mix was prepared with the FastStart Essential DNA Probe Master kit and Universal Probe Library probes according to the manufacturer’s protocols (Roche). PCR was performed in triplicate using the LightCycler 96 System (Roche) with 45 cycles of amplification and annealing temperature of 60°C for two to three biological replicates. Relative change in gene expression was determined by normalizing against 18S rRNA and comparing test group with control.

### Quantitative RT-PCR (human samples)

RNA was extracted from 23 colorectal adenoma and 10 matched normal mucosa samples from 10 different patients diagnosed with FAP using Trizol (Invitrogen). cDNA was synthesized using cDNA Reverse Transcription Kit (Applied Biosystems). Quantitative PCR was performed using SYBR Green Mastermix (Applied Biosystems). CNOT3 specific primers have been previously reported [40] and β-actin was used as endogenous control. Experiments were performed in triplicates and the relative expression was calculated by the ΔCt method using RNA from HCT116 as a reference.

### Bioinformatics analyses

The publicly available cancer database COSMIC was used to identify *CNOT3* point mutations in tumor samples [20]. cBIOPORTAL was used to identify *CNOT3* mRNA mutations from publicly available databases [19]. The USCS Genome Browser was used to visualize the transcription and regulatory elements in the human *CNOT3* chromosome 19 region data from the ENCODE Project (GrCh37 Assembly) [26, 27]. Pairwise global and local protein sequence alignment was performed using programs available from The European Bioinformatics Institute (EBI) [41]. Mutassessor and Polyphen-2 were used to predict possible functional effect of CNOT3 amino acid changes [42, 43].

### Subjects and samples

Targeted sequencing (AmpliSeq) was performed in 37 colorectal adenomas and matched normal mucosa samples from 14 patients with FAP collected at MD Anderson (Supplementary Tables 2 and 3). Informed consent was obtained from all individuals and the Institutional Review Board approved this study. Tissues were retrieved from the endoscopy suite and immediately flash-frozen or preserved in RNAlater (Life Technologies) and then stored at −80° C according to internal protocols. Blood was collected in EDTA tubes and stored appropriately for subsequent extraction of germline DNA. Genomic DNA was extracted from whole blood using the Blood & Cell Culture DNA Mini Kit (Qiagen) and from tissues using the QIAmp DNA Mini Kit (Qiagen). Confirmation of the diagnosis of adenomatous polyps was performed by an expert gastrointestinal pathologist (M.W.T.) in all of those biopsied that rendered enough tissue for both nucleic acid extraction and pathology confirmation. Evidence of high-grade dysplasia could not be verified in all of the samples due to the limited availability of representative tissue material.

### Ampliseq sequencing

Ampliseq sequencing was performed using the next-generation sequencing platform IT Personal Genome Machine (PGM; Life Technologies) by the Sequencing and Non-Coding RNA Program at MD Anderson, using the Ion PGM 200 Sequencing Kit on an Ion 318 Chip Kit (Life Technologies). A multiplex custom targeted gene panel was designed with the Ion AmpliSeq Designer and included the entire coding sequence of *CNOT3* (2,432 base pair, 95% coverage of the exonic sequence). IT Variant Caller v4.2 was run in the somatic low stringency proton mode to detect variants against hg19 on each adenoma and normal BAM file. Then, normal variants were subtracted from matched adenoma variants to create a list of somatic candidates for each adenoma and normal pair. Events located within the first and last 15% of the bases of the read were excluded. Then, a list of somatic candidates went through the following quality control steps: 1) Mutation allele frequencies were re-evaluated after removing variant reads where the mutation lies within the first 15% or last 15% of the bases of the reads; 2) Mutations with more than 2 variant alleles were excluded; 3) Mutations must be covered by a minimum of 100 reads. If a mutation allele frequency is 2-5%, at least 10 reads must show the variant allele. If a mutation allele frequency >5%, at least 25 reads must show the variant allele. Finally, the candidates were imported into a database by vtools [44], which included 5 different functional *in silico* prediction analysis by Polyphen2, SIFT, Mutation Taster, Mutation Assessor and Condel, and annotated with ANNOVAR [45].

### Study approval

The use of zebrafish in these studies was in accordance with an approved IACUC protocol (#17-03) and within institutional guidelines.

### Statistics

Statistical tests used are reported in the figure legends, where all data presented indicate mean ± SEM, unless otherwise specified. A *p*-value less than 0.05 was considered significant. All representative experiments were conducted on 2–3 separate occasions with a minimum of 3 individual samples. All statistical analyses were conducted using Graphpad Prism v 7.02 (GraphPad Software, Inc.).

## SUPPLEMENTARY MATERIALS



## Abbreviations

FAP: Familial Adenomatous Polyposis
CNOT3: CCR4-NOT Subunit 3
APC: Adenomatous Polyposis Coli
RDH: Retinol Dehydrogenase
CRC: Colorectal Cancer
CtBP1: C-Terminal Binding Protein
