# Supplementary material for: Functional characterization of CNOT3 variants identified in familial adenomatous polyposis adenomas
Source: Oncotarget. 2019 Jun 11;10(39):3939–51. doi: 10.18632/oncotarget.27003 (PMC6570471; doi:10.18632/oncotarget.27003)
Supplement: Supplementary file 1 [file oncotarget-10-3939-s001.pdf]

Functional characterization of *CNOT3* variants identified in familial adenomatous polyposis adenomas

SUPPLEMENTARY MATERIALS

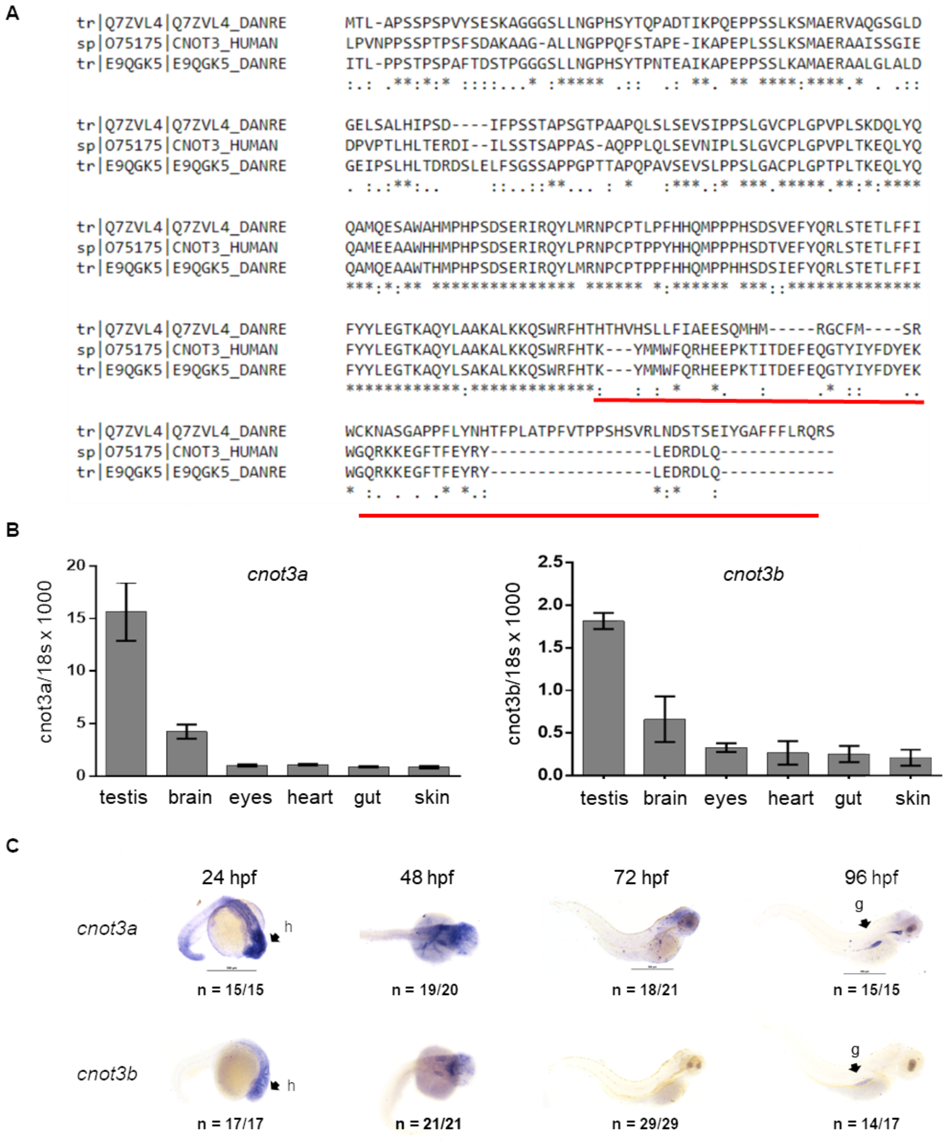

Supplementary Figure 1: Zebrafish cnot3a is the relevant CNOT3 orthologue. (**A**) The C-terminal region (aa 500-753) alignment output from EBI CLUSTAL multiple sequence alignment of hCNOT3 (ID:075175), cnot3a (ID:E9QGK5), and cnot3b (ID:Q72VL4) showing that the cnot3b amino acid sequence diverges from hCNOT3 and cnot3a near the C-terminal end (red lines). (**B**) Expression of zebrafish *cnot3* in adult zebrafish tissues. The *cnot3* genes show similar expression pattern. Values represent mean ± SD. Graph shown above is representative of at 2 independent pooled tissue samples (3 technical replicates each). (**C**). Whole organism *in situ* hybridization (WISH) of zebrafish embryos at 24, 48, 72, and 96 hpf (n > 10 per group). Expression of *cnot3* is observed in the head (h) at 24 hpf and in the gut (g) at 96 hpf.

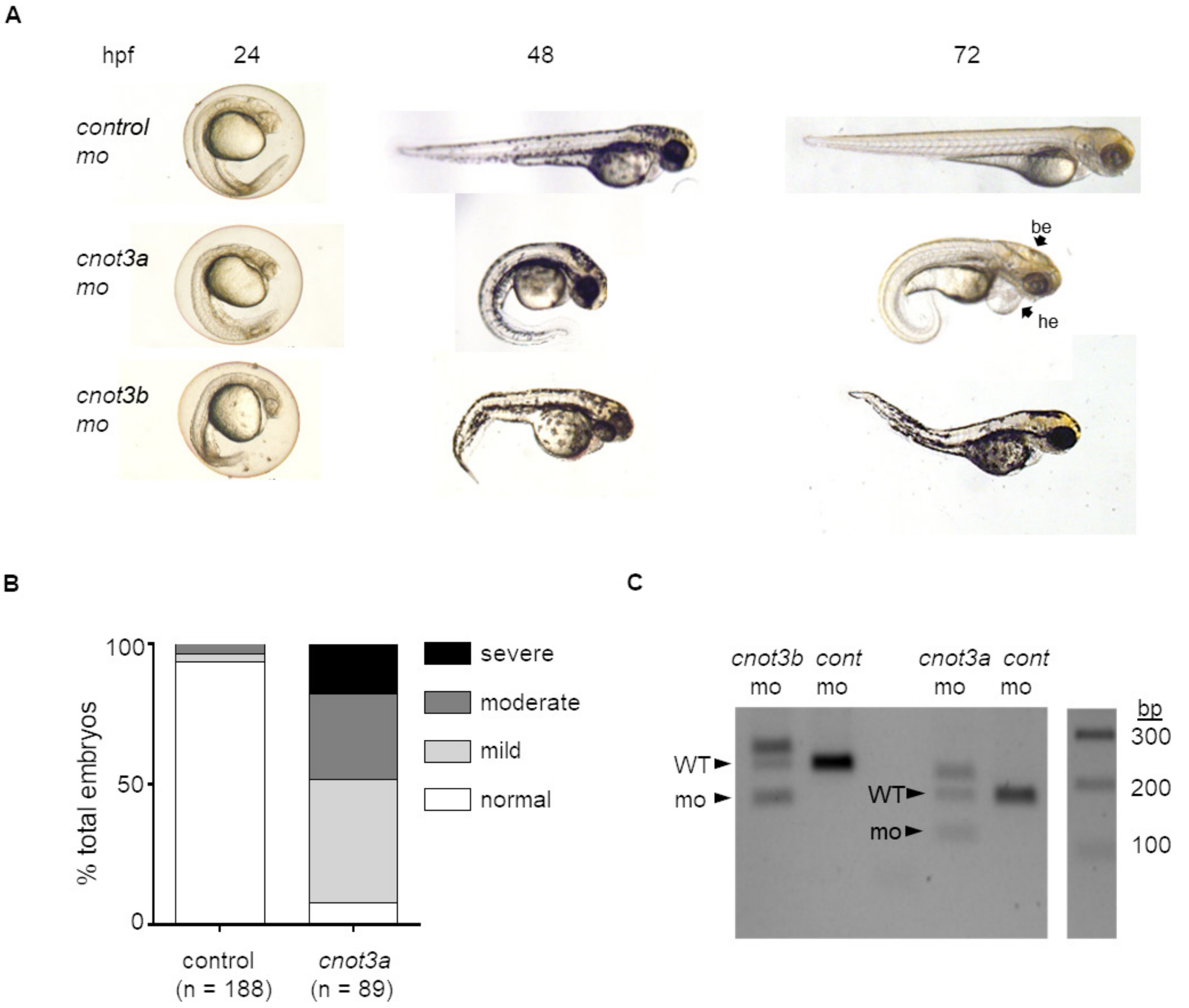

Supplementary Figure 2: Knockdown of *cnot3* gene expression in zebrafish embryos results in head and body developmental defects. (**A**) Time course development of control, *cnot3a*, and *cnot3b* mo injected zebrafish embryos. Zebrafish *cnot3* deficiency leads to lordosis at 48 hpf that persists at 72 hpf for cnot3a knockdown. Knockdown of cnot3a also leads to heart (he) and brain edema (be) at 72 hpf. Depletion of *cnot3b* results in only mild lordosis. (**B**) Graph depicting percentage of *cnot3a* morpholino-injected embryos with observed phenotypic defect/s compared to control mo. Lordosis phenotype is consistently seen in embryos with mild to severe phenotype (>80%). (**C**) PCR of cDNA prepared from 72 hpf control mo or *cnot3* morpholinos. Arrowhead show confirmatory WT and morpholino (mo) bands.

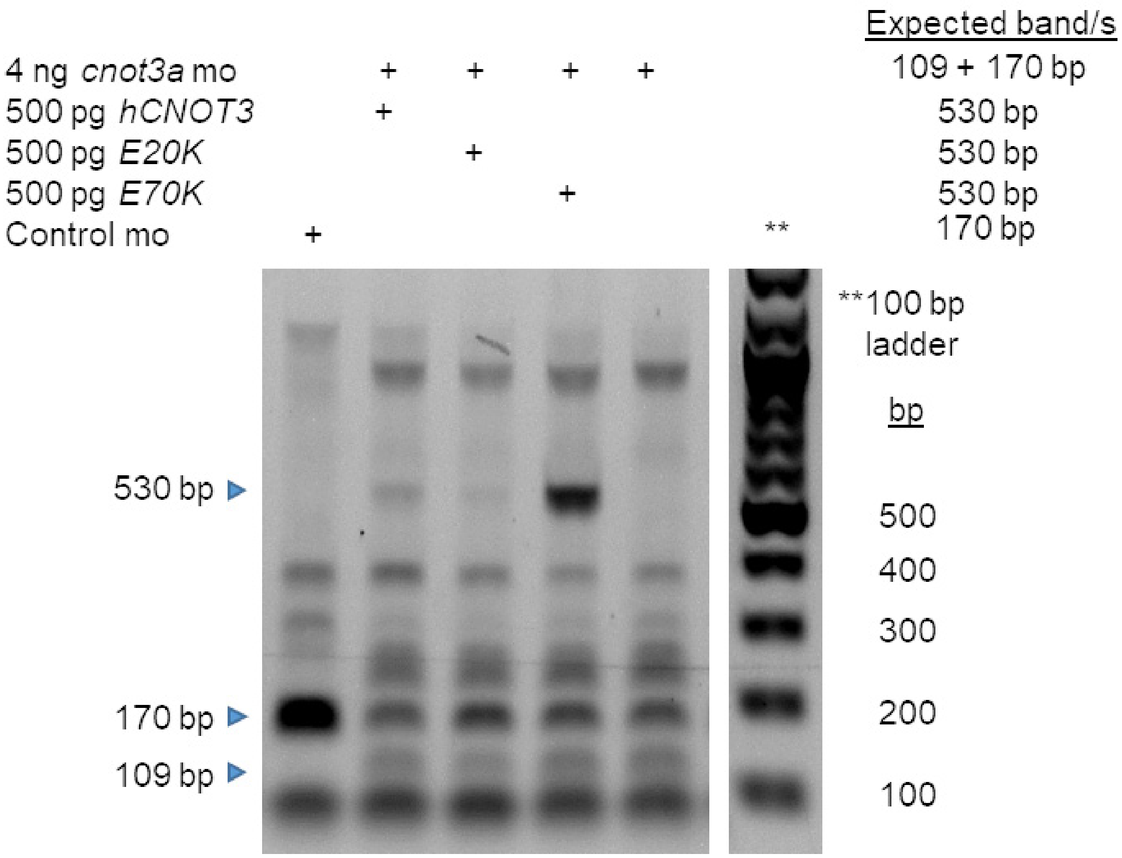

Supplementary Figure 3: Confirmation of *cnot3a* morpholino and *hCNOT3* mRNA injections. Figure shows gel electrophoresis from PCR run using zebrafish embryos injected with the appropriate reagent (+) as cDNA template, and using the confirmatory primers in Supplementary Table 5. Blue arrowheads show the bands - 530 bp (mRNA), 170 bp (*cnot3a*), 109 bp (mo band) – that confirm morpholino and / or mRNA injections.

Supplementary Table 1: European Bioinformatics Institute Global and Local Protein Alignment to compare zebrafish cnot3a and cnot3b amino acid sequence to human CNOT3 protein sequence

	Alignment Score versus Human CNOT3 amino acid sequence	
**EBI TOOL:**	**cnot3a**	**cnot3b**
GLOBAL ALIGNMENT:		
NEEDLE	**2574**	2173.5
STRETCHER	**2286**	1876
LOCAL ALIGNMENT:		
WATER	**2574**	2190
MATCHER	**1970**	1673
LALIGN	**3062**	2626

Larger score means better alignment.

Supplementary Table 2: Clinical characteristics of patients analyzed

Patient			Germline information			Ion Torrent (Target sequencing)
Patient ID	Gender	Age	Gene	cDNA	protein	
**MDAC02**	M	33	*APC*	c.3810T>A	p.Cys1270*	2
**MDAC03**	F	63	*APC*	del exons 11-12	p.?	3
**MDAC10**	F	22	*APC*	c.3440dupA	p.Ser1148Thrfs*18	1
**MDAC14**	F	40	*APC*	del 8-9	p.?	2
**MDAC17**	M	37	*APC*	c.1658G>A	p.Trp553*	2
**MDAC18**	M	27	*APC*	c.4393_4394del	p.Ser1465Trpfs*3	1
**MDAC20**	M	65	*APC*	c.477C>G	p.Tyr159*	1
**MDAC21**	F	70	*APC*	c.487C>T	p.Gln163*	1
**MDAC24**	F	25	*APC*	c.4733_4734del	p.Cys1578Tyrfs*12	5
**MDAC26**	F	28	*APC*	c.847C>T	p.Arg283*	5
**MDAC29**	F	25	*APC*	c.3810T>A	p.Cys1270*	2
**MDAC32**	F	58	*APC*	c.1620insA	p.Gln541Thrfs*19	3
**MDAC33**	M	43	*APC*	c.2894del	p.Asn965Ilefs*15	5
**MDAC34**	M	29	*APC*	c.5936del	p.Asn1979Thrfs*65	4

Abbreviations: M, male; F, female.

Supplementary Table 3: Background information and histopathology of FAP adenoma samples

Sample ID	Location	Type of Polyp	Size	Sample ID	Location	Type of Polyp	Size
MDAC02_P02	Rectum	N/A	N/A	MDAC26_P02	Rectum	N/A	< 5 mm
MDAC02_P03	Rectum	N/A	N/A	MDAC26_P03	Rectum	N/A	< 5 mm
MDAC03_P03	Rectum	N/A	> 5 mm	MDAC26_P04	Rectum	N/A	< 5 mm
MDAC03_P01	Rectum	N/A	> 5 mm	MDAC26_P05	Rectum	N/A	< 5 mm
MDAC03_P02	Rectum	N/A	> 5 mm	MDAC29_P01	N/A	Tubular Adenoma	> 5 mm
MDAC10_P02	Rectum	Tubular Adenoma	< 5 mm	MDAC29_P02	N/A	Tubular Adenoma with HGD	> 5 mm
MDAC14_P04	Rectum	N/A	< 5 mm	MDAC32_P03	Ascending colon	Tubular Adenoma	< 5 mm
MDAC14_P05	Rectum	N/A	< 5 mm	MDAC32_P01	Ascending colon	Tubular Adenoma	< 5 mm
MDAC17_P03	Rectum	Tubular Adenoma	< 5 mm	MDAC32_P02	Sigmoid	Tubular Adenoma	< 5 mm
MDAC17_P04	Rectum	Tubular Adenoma	< 5 mm	MDAC33_P03	Rectosigmoid	N/A	N/A
MDAC18_P02	Rectum	N/A	> 5 mm	MDAC33_P01	Ascending colon	N/A	N/A
MDAC20_P02	Rectum	Tubular Adenoma	< 5 mm	MDAC33_P04	Ascending colon	N/A	N/A
MDAC21_P01	Rectum	Tubular Adenoma	< 5 mm	MDAC33_P05	Ascending colon	N/A	N/A
MDAC24_P01	Rectum	Tubular Adenoma	< 5 mm	MDAC33_P02	Ascending colon	N/A	N/A
MDAC24_P02	Sigmoid	Tubular Adenoma	< 5 mm	MDAC34_P02	Ascending colon	Tubular Adenoma	< 5 mm
MDAC24_P03	Transverse colon	Tubular Adenoma	< 5 mm	MDAC34_P03	Splenic flexure	Hyperplastic polyp	< 5 mm
MDAC24_P04	Hepatic flexure	Tubular Adenoma	< 5 mm	MDAC34_P01	Descending colon	Tubular Adenoma	< 5 mm
MDAC24_P05	Transverse colon	Tubular Adenoma	< 5 mm	MDAC34_P04	Sigmoid	Tubular Adenoma	< 5 mm
MDAC26_P01	Rectum	N/A	< 5 mm				

Supplementary Table 4: Summary of detected CNOT3 mutations using the ion torrent platform and their functional *in silico* analysis

chr	hg19 pos	ref	alt	region type	gene	mutation type	Uniprot aa pos	aa ref	aa alt	PhyloP	SIFT	Polyphen2 HDIV
19	54647842	A	C	exonic	CNOT3	nonsynonymous SNV	120	E	A	1.998	0.56	D;D;D
19	54650354	T	G	exonic	CNOT3	nonsynonymous SNV	.	I	R	0.144	.	.
19	54650355	A	G	exonic	CNOT3	nonsynonymous SNV	286	K	E	2.043	0.74	D;D;D
19	54650355	A	G	exonic	CNOT3	nonsynonymous SNV	286	K	E	2.043	0.74	D;D;D
19	54650355	A	G	exonic	CNOT3	nonsynonymous SNV	286	K	E	2.043	0.74	D;D;D
19	54650355	A	G	exonic	CNOT3	nonsynonymous SNV	286	K	E	2.043	0.74	D;D;D
19	54653350	-	C	exonic	CNOT3	frameshift insertion	.	.	.	.	.	.

chr	hg19 pos	Polyphen2 HVAR	LRT	Mutation Taster	Mutation Assessor	Sample ID	Frequency	Annovar change	Depth
19	54647842	D;D;D	D	D	L	MDAC14_ P05	0.037145	CNOT3:NM_014516:exon6:c. A359C:p.E120A	5840
19	54650354	.	.	D	N	MDAC34_ P04	0.027923	CNOT3:NM_014516:exon10:c. T855G:p.D285E	563
19	54650355	D;D;D	D	D	N	MDAC24_ P02	0.043937	CNOT3:NM_014516:exon10:c. A856G:p.K286E	531
19	54650355	D;D;D	D	D	N	MDAC29_ P01	0.040477	CNOT3:NM_014516:exon10:c. A856G:p.K286E	1789
19	54650355	D;D;D	D	D	N	MDAC32_ P01	0.059016	CNOT3:NM_014516:exon10:c. A856G:p.K286E	570
19	54650355	D;D;D	D	D	N	MDAC34_ P02	0.074935	CNOT3:NM_014516:exon10:c. A856G:p.K286E	724
19	54653350	.	.	.	.	MDAC24_ P01	0.12126	CNOT3:NM_014516:exon13:c .1462_1463insC:p.G488fs	629

Abbreviations: chr, chromosome; hg19 pos, human genome position; ref, reference, alt, alteration; AA, amino acid; SNV, single nucleotide polymorphism; N, neutral; D, probably damaging, B, benign, P, possibly damaging; H, higher impact; M, medium impact: L, lower impact.

Supplementary Table 5: Morpholino and primer sequences

MORPHOLINOS	5′ - 3′ sequence
cnot3a	GTTACACCGTGTTGTCTTACCTTGA
ctbp1	TCCTTATGACGATACCTTCTCATGG
cnot3b	TTCTCCTGTTACCATACCTTGAAGT
**PCR PRIMERS**	**5′ - 3′ sequence**
cnot3a F	GGTCCTGGCTGACACAC
cnot3a R	GTCTTCAAACTGTTCGACGC
CNOT3 F	CACCAAGGAGCAGCTCTATC
CNOT3 R	CCTTATCGTCGTCATCCTTG
cnot3b F	TGAAAAGTTGGAGGAATCGTG
cnot3b R	TTTCTTCATGCCTTCTTCTGC
**RT-PCR PRIMERS**	**5′ - 3′ sequence**
cnot3a F	CGTCCGATTCAGAGAGAATCA
cnot3a R	GAATGATGTGGGGGCATCT
cnot3b F	TGAAAAGTTGGAGGAATCGTG
cnot3b R	TTTCTTCATGCCTTCTTCTGC
β-ACTIN_F	CCCAGAGCAAGAGAGGTATC
β-ACTIN_R	AGAGCATAGCCCTCGTAGAT
CNOT3_RT_F	CGCAAATGGAACGGTTCAAAG
CNOT3_RT_R	GTATTCGTGAGCCACTGGCCA
